# Optimization of Chinese land spatial pattern in the transformation process of resource-based cities: a case study in Tongling City, China

**DOI:** 10.1038/s41598-024-53546-7

**Published:** 2024-03-13

**Authors:** Yun Ye, Yan Qin, Ran Yu, Qun Wu

**Affiliations:** 1 College of Public Administration, Nanjing Agricultural University, Nanjing, 210095 China; 2 China Institute of Resources, Environment and Development, Nanjing Agricultural University, Nanjing, 210095 China; 3 College of Economics and Management, Anhui Agricultural University, Hefei, 230036 China

**Keywords:** Environmental sciences, Environmental social sciences

## Abstract

Optimizing the spatial layout of the national territory is crucial for realizing the transformation and development of resource-oriented cities in the context of high-quality development in the new period. This paper takes Tongling City as a case study, based on the analysis of the historical development foundation, then uses the SD-FLUS comprehensive model to carry out a systematic analysis of the transformation in five dimensions of economy, society, population, science and technology, resources and environment, and discusses the optimization of spatial pattern under the contextual simulation. The conclusions are as follows: ① The hierarchical framework of “system-indicator-element” is not only internally interconnected, but also inextricably linked with the relationship between the various categories of the land use system. ② Decrease of cropland, forest, water, grassland, and barren decreases from the economic development, social progress, and comprehensive development, and there is a small increase in the area under the scenario of resource and environmental protection, and the direction of the change of the impervious is in the opposite direction. ③ Cultivated land is retained in situ and concentrated to a small extent, forested land is reduced to a small extent while the status quo is maintained, and the Yangtze River water system will be retained and protected to a large extent, but part of the waters of Zongyang County will give way to the expansion of construction land under the development objectives of the new county. Building land will be expanded and extended to the northeast in the original site area, while the southwest corner of the original county center will be expanded to some extent in Zongyang County to promote the county’s economic development.

## Introduction

Resource-oriented cities are cities in which the extraction and processing of natural resources such as minerals and forests in the region are the leading industries, and they are cities that are important guarantors of the security of the country’s resources and energy, and whose state of development and results of transformation are of significant effectiveness. The emergence of many problems such as lack of resources, environmental degradation, and lack of economic development power indicates the urgent need for a new stage of transformation and development based on the special characteristics of development. The study of resource-based cities has a historical stage, since the industrial revolution in the 1860s, there have been many cities and regions around the world that mainly focus on resource extraction and development. After the 1920s, the demand for coal, minerals, oil, and other resources rose in various places, and large-scale industrial production and urbanization pushed forward the economic development, but also led to the further development of resource-based cities and regions. The resource-based cities and regions have been further developed. During this period, the Canadian scholar Innis’s monograph *An Introduction to Canadian Economic History*, opened the prelude to the study of resources and urban development^1^. Then, the economic growth theory proposed by Melville^2^, the four-stage life cycle theory of construction, employment, transition, and maturity proposed by Lucas^3^, one step further, the six-stage life cycle theory of Bradbury’s expansion of the decline stage and the closure stage^4^ laid the theoretical foundation for the study of the development of resource-based cities. With the great innovation of production mode brought by the third industrial revolution, the research perspective has been expanded to the global industrial system, foreign trade, labor force, economic structure, etc.^5–8^, and further integrated the theories of institutional economics, environmental economics, development sociology, and other disciplines, strengthening the exploration of the natural environment, cultural preservation, women’s empowerment, regional spatial structure, government intervention, and other elements of the perspective^9,10^.

Compared with western countries, China’s industrialization and urbanization process is relatively late, and so is the formation and development of resource-based cities. In the early stage of the founding NRC, the country implemented the strategy of prioritizing heavy industry, resource-based industries as the essential material support for industrial development, assumed an important role in the country’s construction of large-scale industrialization, therefore, the status of resource-based cities in the national productivity layout is also of great significance. In this context, academic research focuses on the location and construction of industrial bases, industrial development layout, productivity conditions of resource cities, resource evaluation, etc., with a strong feature of the government’s strategic plan. Since the reform and opening in 1978, the national strategy has gradually shifted from a planned economy to a market economy. Resource cities have been oriented to economic growth and plundering resources, which has resulted in many urban problems, such as industrial structure imbalance, economic growth slowdown, unemployment rate increase, and ecological environment deterioration. During this period, the focus of academic attention was centered on industrial structure adjustment, sustainable urban development, and other perspectives^11–13^. After the twenty-first century, the country entered a new stage of development, no longer focusing on the pursuit of economic development speed. Especially the 18th National Congress of the Communist Party of China (CPC), which elevated the concepts of ecological environmental protection, harmony between man and nature, and the integration of the development of numerous disciplines to a new height. Domestic scholars’ research perspectives on resource-based cities have gradually expanded to urban development strategies, transformation and development capabilities, industrial space patterns, ecological environment, and other aspects^14–18^.

The territorial spatial pattern is the overall layout of natural resources, human life, economic environment, social development, and other integrated spaces based on territorial space^19^, which is the core of spatial planning. Spatial planning originated in Europe, since the 1920s, Britain, the United States, Japan, and other developed countries have begun the practice of spatial planning one after another^20^, especially after the 1980s, the worldwide trend of sustainable thinking, promoting the holistic construction of spatial planning and the coordinated development of various elements. Internationally, regional planning^21^, urban planning^22^, land use planning^23^, landscape planning^24^, and habitat planning^25^ have been taken as research objects, and studies have been carried out on perspectives such as optimization of urban green space^26^, optimization of food security space^27^, and optimization of land use^28^. 2019, China (CPC) Central Committee published *Opinions on Establishing a System of Territorial Spatial Planning and Supervising Its Implementation* (hereinafter *Opinions*), which consolidated multiple types of planning in the historical period and constructed a general framework for China’s territorial spatial planning. Territorial spatial planning is a major innovation in the field of spatial planning in China, combining macro-strategy and practicability. Domestic scholars’ research on national spatial planning mainly focuses on the theoretical connotation^29^, planning implementation^30^, spatial optimization^31^, and other aspects.

Resource-oriented cities have specific spatial problems due to their development characteristics, such as the geological and geomorphological problems of mine pits and coal processing plants, the low quality of public space, the problem of low utility land and idle land, etc.^32^. In this regard, the transformation and development of resource-oriented cities should enhance the importance of spatial patterns. Therefore, the transformation and development of resource-oriented cities should pay more attention to the spatial pattern. Academic research on this issue focuses on the impact of changes in the land use structure and layout of resource-based cities on the ecological environment^33^, sustainable development^34^, multifunctional carrying capacity^35^, urban security elasticity^36^, and other aspects. Tingting He et al. used nighttime light remote sensing data to identify the transformation of spatial patterns in resource-based cities in China and found that the land use expansion of most resource-based cities has been shrinking in recent years^37^. When monitoring the surface temperature of resource cities, Wei Chen et, al. found that the expansion of built-up area would increase the heating effect of surface temperature^38^. Expansion of the built-up land area in resource cities not only increases the surface temperature and heat island effect, but also affects the local ecological environment due to the attenuation of ecological land such as forest land, grassland, and watershed^39^. Therefore, Jiazheng Han et, al. conducted a spatial and temporal evolution analysis of the value of ecosystem services for resource-based cities and analyzed the future trend, and the results of the study can provide ideas and references for environmental protection and green sustainable development planning in resource-based cities^40^.

To summarize, the transformation of resource-based cities focuses on the internal quality improvement of resources and resource industries themselves in line with the product life cycle on the one hand, and on the other hand, according to the current problems brought by historical development factors, such as population migration, policy target bias, ecological environment sacrifice, etc., and in combination with the objectives of upgrading the industrial structure, controlling the energy consumption, and green low-carbon development, the transformation and upgrading in terms of policy coordination, facility support and cultural concepts are carried out. The transformation and upgrading of policies, facilities, and cultural concepts have been carried out. At the same time, it is a general trend to integrate spatial optimization into the transformation process and promote the formation of a harmonious social spatial network of resource cities with common governance and sharing^41^. Although the current research on resource-based cities and national land spatial optimization has made great progress, there are still directions that can be broadened: (1) In the research on the transformation of resource-based cities, many scholars focused on one or several perspectives, and few studies were conducted from the perspective of systemic relevance, with the combination of multi-systems and multi-factors. (2) In the process of optimizing the spatial structure of the national territory, there were few specific analyses for resource cities, a research unit with a unique development history.

Therefore, this paper will start from the background of the transformation of resource cities, comprehensively consider the multi-systems of economy, society, population, science and technology, and resource and environment, and simultaneously set up five scenarios of natural tendency (NT), economic development (ED), social progress (SP), protection of resources and environment (PRE), and coordinated development (CD), to jointly construct a multi-systems and multi-situations optimization scheme of land use structure and layout in the context of transformation and development. This study will fill the above gap by addressing the following three questions:

1. What systems and factors jointly influence the process of transformation and development of resource-based cities?
2. What are the differential changes in land use structure and spatial layout in the process of transformation and development of resource-based cities?
3. What feasible predictions and suggestions can be provided for the future development of resource-based cities?

The structural setting of this paper is organized as follows. Part 1 emphasizes the theoretical basis and relevant progress of the transformation and development of resource-oriented cities and the optimal layout of land space. Section “Theoretical Framework” constructs a multi-system analytical framework for the transformation and development of resource-based cities. Materials and methods will be summarized in Section “Materials and methods”. The results and discussion will be presented in Sections “Results and analysis” and “Discussions”. Finally, Section “Conclusions” summarizes the conclusions and presents policy recommendations.

## Theoretical framework

The transformation of resource cities is a complex integrated system, which requires the interaction of various subsystems and their intrinsic factors to jointly promote, and this paper explores five subsystems, namely, economic, social, demographic, scientific and technological, and resource environment. The level of economic development is a key indicator of whether a resource city can have the development momentum, usually characterized by the GDP factor, and various types of land will be dependent on the main industries, which will bring the corresponding industrial output and product output, so this paper chooses this as the key factor of the economic subsystem to conduct the land use system. The social quality of resource-based cities is not only affected by economic development but also by other social configurations of urban space, such as infrastructure configuration and social security level. In the early stage of development, resource-based cities will focus on the construction of industrial zones, and then whether to carry out balanced urban development needs to be explored, and the construction of land as the main affected land category is in line with the expansion of urban development. At the same time, it is also worthwhile to investigate whether there is a historical increase or decrease in the amount of forest land and waters due to resource exploitation in the early stage and ecological restoration projects in the later stage. The level of education, as an important part of the social subsystem, is interrelated with the population subsystem and the science and technology subsystem, and science and technology, talents, and innovation power are taken as the productivity, resources, and driving force for the transformation and development of resource cities, which help to transform the industrial structure of resource cities from rough and inefficient to high-quality and efficient. Due to the long time and high-intensity mining and excavation of resources, resource cities generally face serious environmental pollution and ecological damage and other problems, such as over-exploitation leading to surface subsidence, mountain mining leading to the destruction of vegetation, etc., which not only affects the number of ecological land in the land-use system such as forest land, grassland, waters, etc. but also causes different degrees of damage and pollution to it. Therefore, it is necessary to set up the resource environment subsystem in the transformation process of resource cities, to gradually restore the area and ecological original appearance of woodland, watershed, grassland, and other land categories, to make them play the ecological efficacy of water nourishment, soil and water preservation, air purification, and temperature regulation, and to promote the green and ecological transformation of resource cities, and to gradually form a coordinated and sustainable development path.

**Figure 1:**
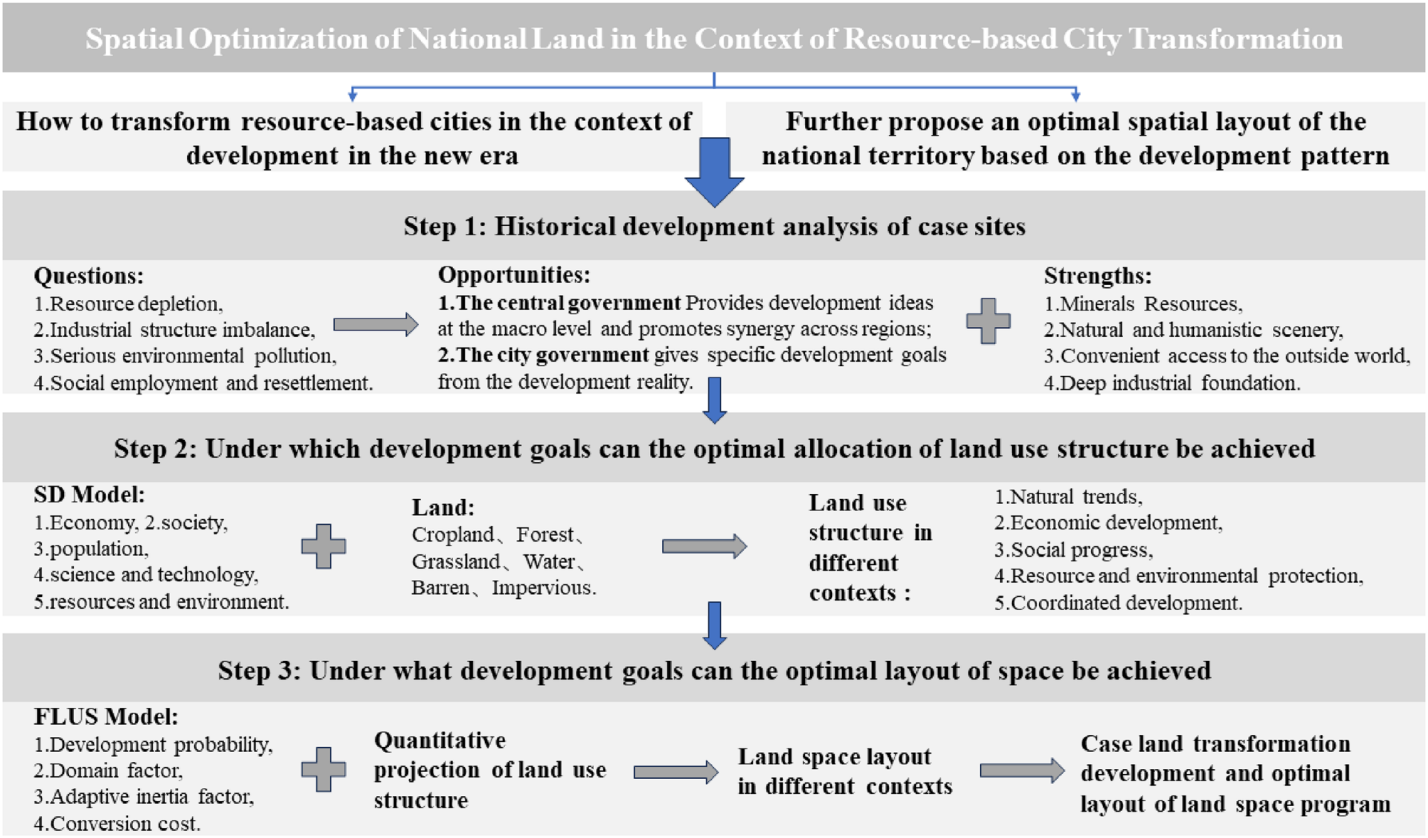
Workflow of our study.

Based on the above theoretical analysis, this paper divides the issue of land space optimization in the context of the transformation of resource cities into two parts to carry out research. The first one is how resource cities can carry out transformation and development in line with the development of the new era, The second one is to further put forward the optimization of the layout of the land space in line with the development of resource cities based on the development vein of resource cities. To realize the above objectives, this paper will carry out the following stages of research (Fig. 1). First, based on the advantages of Tongling’s development foundation, it meets the new challenges of transformation and development under the opportunities of the current policy bias. Secondly, based on the SD model, Tongling’s transformation and development is divided into five key aspects, this paper draws structural land use predictions under different circumstances and intends to judge under what development goals resource cities can realize the optimal allocation of land use structure. Thirdly, with the help of the spatial evolution function of the FLUS model, this paper explores the mutually reinforcing relationship between the rational allocation of land use structure and the optimization of spatial layout. Finally, combining the prediction results of the land use quantitative structure of the SD model with the spatial layout simulation under different situations, and intending to further deduce the development objectives under which the resource-oriented city can realize the optimal spatial layout.

## Materials and methods

### Research area

**Figure 2:**
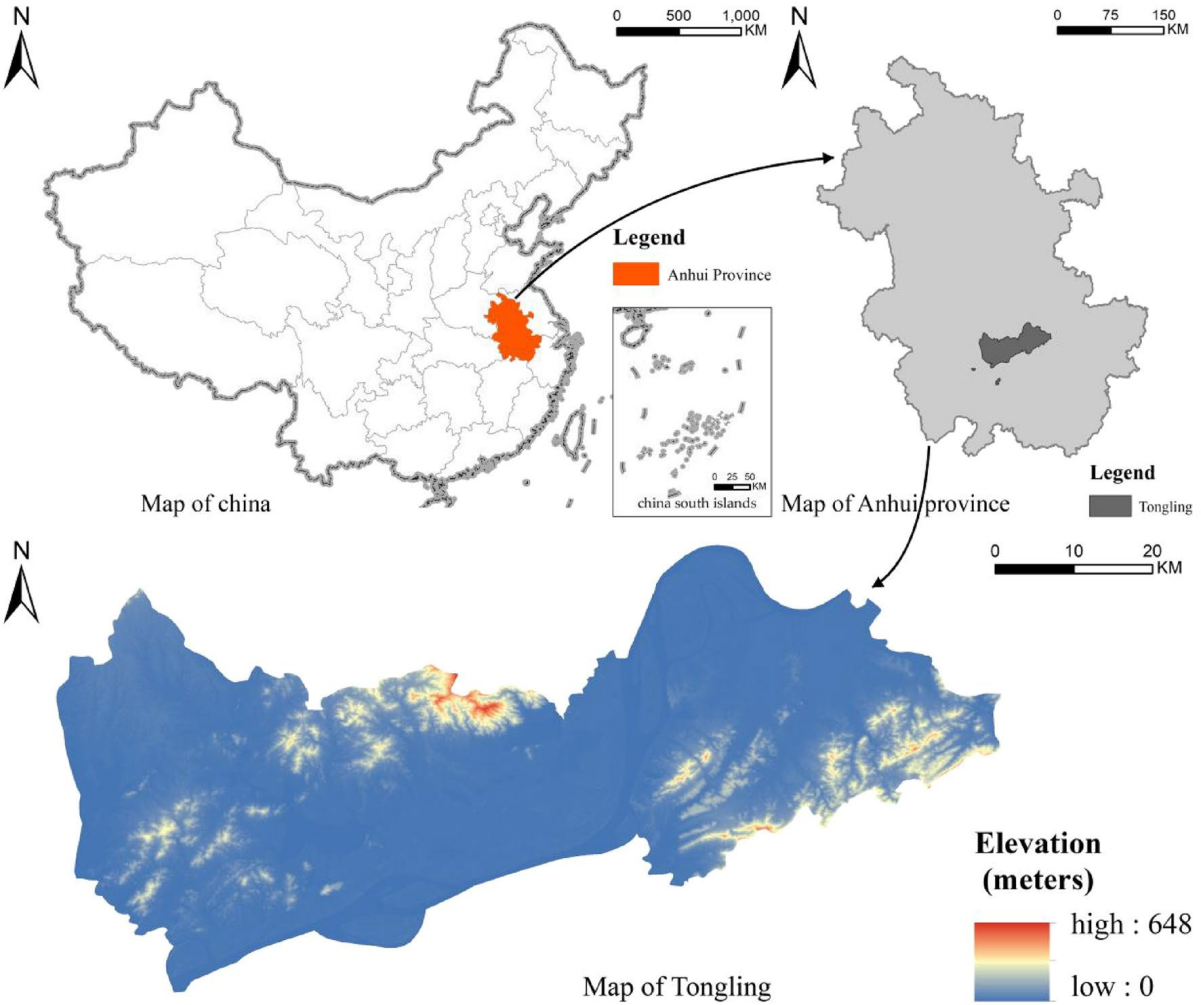
Tongling City district map. *Note*: Revision number is GS (2020)4619, no modifications to the base map, URL: http://bzdt.ch.mnr.gov.cn/index.html. This diagram was created by the software GIS 10.6, URL: https://desktop.arcgis.com/zh-cn/index.html.

Tongling (117° 04′–118° 09′ E, 30° 38′–31° 09′ N) is a prefecture-level city under the jurisdiction of Anhui Province, located in East China, with three districts (Tong guan District, Yian District, and Suburban District) and Zongyang County under its jurisdiction (Fig. 2). In terms of natural environment, it has a humid monsoon climate in northern subtropics, with low mountains and hills in the southern part of the city, the Yangtze River passing through the city, a high density of water network, long rivers and ditches, and extensive lakes and swamps. Socio-economic aspects, as of the end of 2021, the resident population of 1.306 million people, the urbanization rate of 66.3%, the gross regional product of 116.56 billion yuan, the structure of the three industries is 5.1: 49.5: 45.4.

Tongling has some disadvantages at this stage of development, but its unique advantageous conditions and opportunities of the new era can promote the completion of the transformation challenges. At present, Tongling is accompanied by resource depletion brought about by the gradual approaching of the upper limit of resource exploration reserves, industrial structure imbalance brought about by the excessive dependence on industry for economic development, serious environmental pollution brought about by the process of mining mineral resources, and industrial transformation brought about by industrial unemployment and resettlement of outstanding social problems and other development constraints. However, Tongling is located in the Yangtze River copper and iron mineralization belt, the distribution of mineral resources is brought about by the concentration of resource advantages, and it has beautiful natural scenery, copper mining has a long history, brought about by the natural and humanistic advantages; highway, railroad, water transport lines of the construction of the external transportation advantages; Tongling Nonferrous Metals Company for the resource-based industries in Tongling, laying a deep industrial foundation and socio-economic advantages. At the same time, the attention and importance of governments at all levels are the development opportunities in the new era. The central government has not only given Tongling multiple positions but also promoted its integration into regional development, provided development ideas at the macro level, and promoted the synergistic development of various regions. The municipal government, starting from the actual development, proposed that the copper processing industry is the industry with the most growth potential in the city’s industrial economy, and to further promote the rapid and healthy development of the copper processing industry in Tongling, it should strongly guide and support enterprises to implement technological transformation and innovation and to promote the continuous optimization and upgrading of the copper industry structure.

### Research methodology

#### SD model

System dynamics models are used to analyze information feedback systems and study the internal dynamic structure and feedback mechanisms. Since it was proposed in the 1950s, it has been widely used in many fields. The modeling process is mainly constituted by clarifying the modeling objectives, determining the system boundary, constructing causal relationships, constructing the model, model testing and revision, and analyzing the simulation results. The spatial boundary of the system identified in this paper is the city area of Tongling (excluding the two administrative enclaves), the time step is set to 1 year, and the time of historical data for model testing is 2005–2020, and the forecast years are 2021–2035. By determining the model parameters, extracting the parameter values in the data, and applying linear regression, table function, arithmetic average, and gray prediction methods, the mathematical equations between the variables are constructed to further describe the relationship between the model variables.

Through the relative error calculation, the historical real data of 2005–2020 in the system dynamics model of the transformation of resource-oriented cities in Tongling and the results of the model equations simulating 2005–2020 are tested and corrected, and the arithmetic method is shown in Eq. (1).

1 $$ \begin{array}{*{20}c} {\theta = \frac{{\left| {A - A^{*} } \right|}}{A} \times 100\% } \\ \end{array} $$

where θ is the relative error, reflecting the degree of confidence in the simulated value of the model; A is the real value, and $A^{*}$ is the simulated value corresponding to it.

The system dynamics model constitutes different simulation situations according to the changes in the values and combinations of different factors. In this paper, we refer to the *National Sustainable Development Plan for Resource Cities (2013–2020)*, *Outline of the 14th Five-Year Plan and 2035 Vision of National Economic and Social Development of Anhui Province*, *Outline of the 14th Five-Year Plan and 2035 Vision of National Economic and Social Development of Tongling City*, *Overall Land Utilization Plan (2006–2020)* and other relevant documents to control the requirements and scope of indicators for urban development and transformation of resource cities. Based on the comprehensive construction of the above indicators, six indicators are selected, GDP growth rate, urbanization rate, social factor, natural factor, pollution control factor, and scientific and technological progress factor. Through the state combination of different indicators, five scenarios of NT, ED, SP, PRE, and CD are set to simulate the land use of resource-oriented cities (Table 1). The state “natural” indicates that the indicator develops according to the general development state, the state “superior” indicates that the indicator develops according to the relatively rapid development state, and the state “average” indicates that the indicator develops according to the general development state. The state “average” indicates that the indicator has developed according to the average development state of the general development state and the relatively rapid development state.

**Table 1:** Context setting description table.

Contextualization	GDP growth rate	Urbanization rate	Social factor	Natural factor	Pollution control factor	Technological progress factor
Natural trends	Natural	Natural	Natural	Natural	Natural	Natural
Economic development	Superior	Natural	Natural	Natural	Natural	Superior
Social progress	Natural	Superior	Superior	Natural	Natural	Natural
Resource and environmental protection	Natural	Natural	Natural	Superior	Superior	Natural
Coordinated development	Average	Average	Average	Average	Average	Average

Based on the above state classification, each context setting is specified. NT scenario serves as a reference scenario for land use change, and each indicator is developed according to the general development state. Among ED scenario, considering that the level of GDP growth and scientific and technological progress will promote economic development, the GDP growth rate, and the scientific and technological progress factor, which consists of the number of scientific research institutes, the number of middle- and high-level scientific researchers, the number of patents granted, and the number of research funds of enterprises, are set as the superior state. Among the SP scenario, according to the evolution of China’s urban development, social progress is still affected by the level of urbanization to a certain extent, and the configuration of healthcare, education, social security, and infrastructure can improve social fairness and people’s sense of well-being, which can reflect the overall level of social progress, so the urbanization rate and the social factor will be set as superior. In the PRE scenario, the economic and social development process of resource-based cities relies heavily on the acquisition of natural resources, and the production behavior and lifestyle of the previous stage inevitably brought about environmental pollution. In the historical period of urban development towards a new stage, especially for the historical legacy of resource exploitation and industrial pollution in resource-based cities, it is more important to strengthen the protection and efficient utilization of resources and the protection of the ecological environment, so the natural factors and pollution control factors are set as superior state. Among CD scenario, the transformation and development of resource-based cities should take into account the systemic nature of urban development and realize coordinated development under the premise of comprehensive consideration of high-quality improvement of economic level, stable and steady social life, and high-quality and livable ecological environment, therefore, this scenario adjusts the development status of the above scenarios to the average value status.

#### FLUS model

In this paper, the development evolution probability of each land use type is simulated using an artificial neural network algorithm using the FLUS (Future Land Use Simulation) model. The calculation formula is as follows:

2 $${\text{T}}{\text{P}}_{\text{p,k}}^{t}={P}_{\text{p,k}}\times {\Omega }_{\text{p,k}}^{t}\times {I}_{k}^{t}\times {\text{s}}{\text{c}}_{c\to k}$$

Where $TP_{p,k}^{t}$ is the overall conversion probability of a p-tuple cell from the original site type to a k-type site at time t;* P*_*p,k*_ denotes the development probability of a tuple *p* converting to a *k* type site; $\Omega_{p,k}^{t}$ denotes the neighborhood influence factor; *It k* denotes the adaptive inertia coefficient; and *sc*_*c→k*_ denotes the cost of conversion of a *c* type site to a *k* type site.

##### Development probability

A random sampling method was used to sample the land use data, then the data of the driving factors were normalized, and finally, the development probability of each class was calculated by the BP-ANN model.

##### Domain impact factor

The neighborhood influence factor reflects the interaction between different land use types in the spatial pattern of the land and between different land use units within the neighborhood in the context of the transformation of resource cities, and the 3 × 3 Moore neighborhood model was selected for calculation in this study:

3 $$ \Omega_{{{\text{p}},k}}^{{\text{t}}} = \frac{{\sum\limits_{3 \times 3} {con\left( {c_{p}^{t - 1} = k} \right)} }}{3 \times 3 - 1} \times w_{k} $$

where: ${\text{con}}\left( {{\text{c}}_{{\text{p}}}^{{{\text{t}} - {1}}} = {\text{k}}} \right)$ denotes the total number of tuples occupied by the site type *k* at the last iteration t − 1; w_k_ denotes the neighborhood factor parameter of each type of site; $\Omega^{{\text{t}}}_{{{\text{p}},{\text{k}}}}$ is defined as the neighborhood influence factor of the tuple *p* at the moment *t*. The neighborhood factor parameter ranges from 0 to 1, with the closer to 1 representing the stronger expansion capacity of the site type.

##### Adaptive inertia coefficient

The adaptive inertia coefficient for each unit is automatically adjusted to the current land type inheritance based on the difference between the expected demand and the actual assigned land type. The adaptive inertia coefficients are defined in the following equation:

4 $$ {\text{I}}_{{\text{k}}}^{{\text{t}}} = \left\{ {\begin{array}{*{20}l} {{\text{I}}_{{\text{k}}}^{{{\text{t}} - {1}}} } \hfill & {\left( {{\text{if }}\left| {{\text{D}}_{{\text{k}}}^{{{\text{t}} - 1}} } \right| \le \left| {{\text{D}}_{{\text{k}}}^{{{\text{t}} - 2}} } \right|} \right)} \hfill \\ {{\text{I}}_{{\text{k}}}^{{{\text{t}} - {1}}} \times \frac{{{\text{D}}_{{\text{k}}}^{{{\text{t}} - 2}} }}{{{\text{D}}_{{\text{k}}}^{{{\text{t}} - 1}} }}} \hfill & {\left( {{\text{if D}}_{{\text{k}}}^{{{\text{t}} - 1}} {\text{ < D}}_{{\text{k}}}^{{{\text{t}} - 2}} { < }0} \right)} \hfill \\ {{\text{I}}_{{\text{k}}}^{{{\text{t}} - {1}}} \times \frac{{{\text{D}}_{{\text{k}}}^{{{\text{t}} - 2}} }}{{{\text{D}}_{{\text{k}}}^{{{\text{t}} - 1}} }}} \hfill & {\left( {{\text{if0 < D}}_{{\text{k}}}^{{{\text{t}} - 1}} {\text{ < D}}_{{\text{k}}}^{{{\text{t}} - 2}} } \right)} \hfill \\ \end{array} } \right. $$

where: $I_{k}^{t}$ refers to the inertia coefficient of land class *k* at iteration time t; $D_{k}^{t - 1}$ denotes the area difference between land demand and allocation at time *t − *1.

The conversion costs can represent the interconversion restriction rules between the site types and, at the same time can be set differentially according to different simulation scenarios.

### Data sources

The data required in this paper are mainly land use/surface cover data, socio-economic statistics of system dynamics elements, and spatial variable data of driving factors affecting land use change, and the data required in this paper are obtained after data collection, screening, and processing (Table 2).

**Table 2:** Data presentation sheet.

Data category	Data name	Description	Data source	Literature resources
Land use/surface cover data	CLCD (China land cover dataset)	30 m × 30 m	http://doi.org/10.5281/zenodo.4417809	^26–28^ ^31^
Socio-economic statistics	China city statistical yearbook	Statistical data	http://www.stats.gov.cn/	^11–18^
	Tongling statistical yearbook	Statistical data	https://tjj.tl.gov.cn/tjnj/	
	Tongling City government work report	Statistical data	https://www.tl.gov.cn/xxgk/zfgzbg/	
	Anqing city statistical yearbook	Statistical data	https://tjj.anqing.gov.cn/tjsj/tjnj/index.html	
	Anqing City government work report	Statistical data	https://www.anqing.gov.cn/tjsj/tjgb/index.html	
Spatial variable data	Elevation	30 m	https://www.resdc.cn/Default.aspx	^32–40^
	Slope	30 m	From the elevation calculation	
	Slope direction	30 m	From the elevation calculation	
	Distance from road	30 m	OpenStreetMap	
	Distance from railroad	30 m	OpenStreetMap	
	Distance from highway	30 m	OpenStreetMap	
	Distance from provincial road	30 m	OpenStreetMap	
	Distance from City Hall	30 m	OpenStreetMap	
	Distance from county (district) government	30 m	OpenStreetMap	

## Results and analysis

### Analysis of the results of the land-use structure simulation

#### Land use structure SD modeling

**Figure 3:**
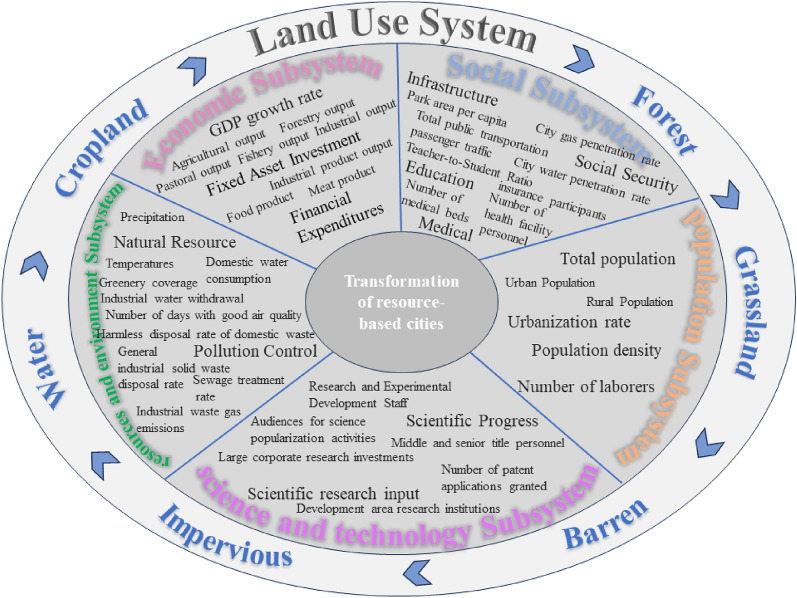
Flow chart of SD model of resource-based city transformation.

**Figure 4:**
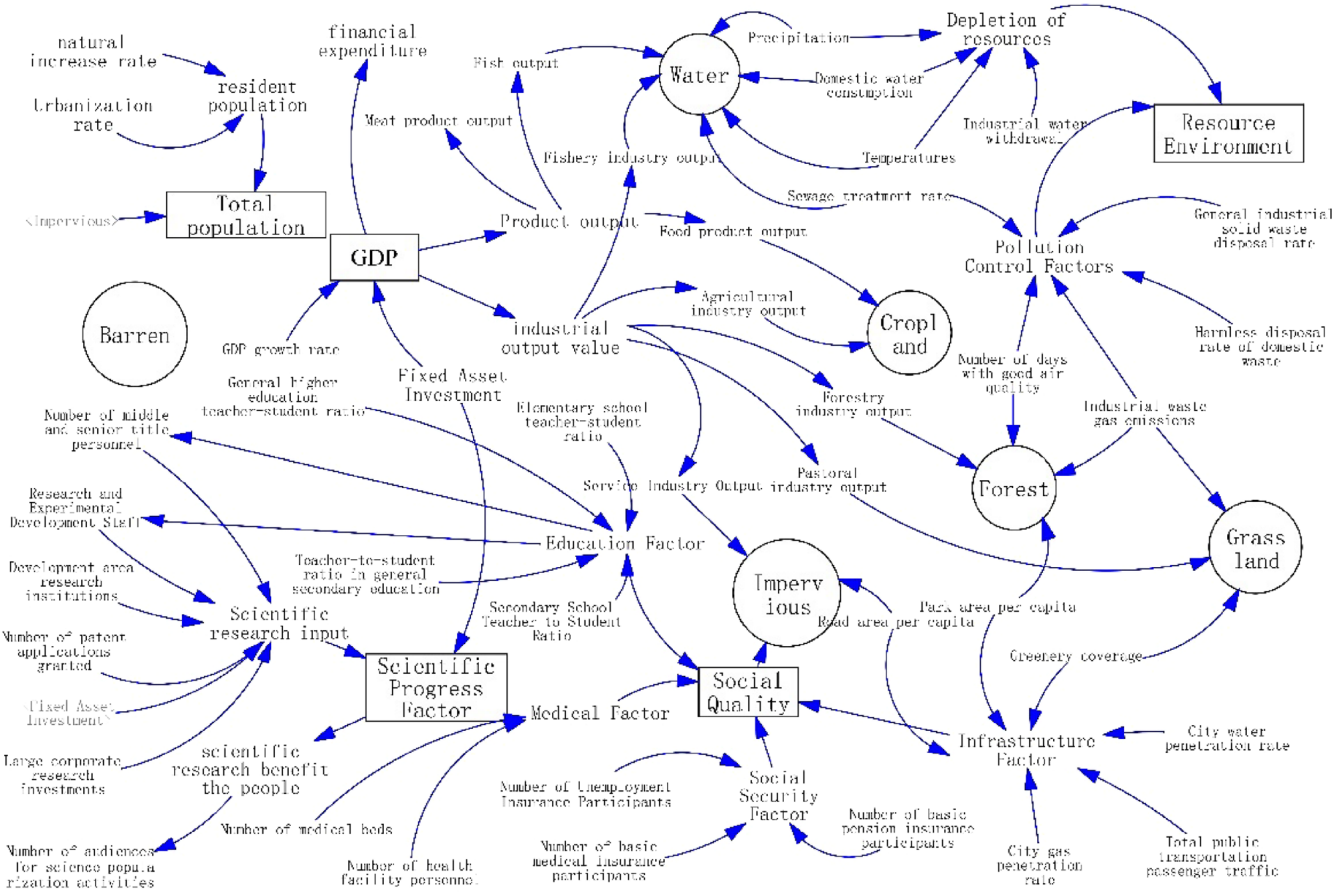
Tongling City transformation system flow chart.

In Fig. 3, the SD model definition explores the interactions between subsystems and combines five aspects of the economy, society, population, science and technology, and resources and environment with the relationship between various types of land use. Figure 4, using Vensim software to draw a flowchart of the system dynamics of the land use structure in the context of the transition of the resource-based city.

① Economic subsystems. An increase in the level of economic development promotes population mobility social progress and development and raises the amount and rate of fiscal expenditure, which can affect the other subsystems. GDP is selected as the state variable in the economic subsystem, and its corresponding flow rate variable is the amount of change in GDP growth, which is determined by the GDP growth rate (GGR). The industrial output value is the important economic efficiency performance of different land use categories, which consists of agricultural output value (AIO), forestry output value (FIO), pastoral output value (PIO), fishery output value (FIO), and service industry output value (SIO). Product output, as an important economic output performance of different land use categories, consists of grain output (FPO), meat product output (MO), fishery product output (FO), and industrial product output (IPO), while financial expenditure (FE), fixed asset investment (FAI) and other elements are also set.
② Social subsystems. As an indispensable social perspective of urban development, social subsystems play an important role as a bridge. In this paper, we set up four dimensions of social quality (SQ), namely, medical factor (MF), infrastructure factor (IF), social security factor (SSF), and education factor (EF), according to the livelihood issues and the key areas of concern of urban development. Among them, the medical factor includes the number of health facility personnel (NHFP) and the number of medical beds (NMB); the infrastructure factor is constructed from the perspectives of energy security for daily life, transportation, and travel convenience, and the human environment, and includes the road area per capita (RAPC), the city gas penetration rate (CGPR), the city water penetration rate (CWPR), the total number of passengers on public transportation (TPRP), and the park area per capita (PAP); the social security factor is set to measure the social quality (SQ) in four aspects. area per capita (PAP); the social security factor is characterized by insurance protection affecting people’s livelihoods, including basic medical insurance (NBMIP), unemployment insurance (NUIP), and basic old-age pension insurance (MBPIP); the education factor takes into account the differences in age groups and levels of education, and is set for elementary school (ESTR), secondary school (SSTR), general secondary and vocational education (GSETR), general higher education teacher-student ratio (GHETR).
③ Population subsystem. As the main body of economic and social development, human beings are the boosters of the whole urban development system, and their daily activities are directly or indirectly related to the subsystems of economy, society, resources and environment, and scientific and technological progress. The population mainly plays a role in different land types such as construction land for urban development and arable land for rural areas, so the urban population (UP) and rural population (RP) are chosen to characterize the population of the spatial region, and the level of urbanization rate (UR) and population density (PD) directly affects the degree of resource consumption, the number and density of education, medical care, infrastructure and other configurations, as well as the labor force (LN) configuration of the region. (LN) allocation, and therefore equally important as a factor in the population subsystem.
④ Science and technology subsystem. The level of scientific and technological development is the key if resource cities want to realize the transformation and development in the new economic era. Scientific and technological progress (SPF) requires an increase in scientific research investment (SRI), while the transformation of scientific and technological achievements will indirectly affect economic development. In this paper, the number of scientific research institutions (DRI), the number of patent applications and authorizations (NPAG), the number of research and experimental development personnel (REDS), the number of middle and senior title personnel (NMSP), and the research capital investment (LCRI) of large enterprises in the development zones are taken as the important factors of scientific research investment. At the same time, scientific and technological progress will not only promote the development of the economy but also indirectly act on the progress of the society, for example, the beneficiary effect such as the improvement of the level of education and the improvement of social convenience, which feature is characterized by the number of audiences of science popularization activities (NASP).
⑤ Resource environment subsystem. Natural resources are an important material basis for sustaining human social activities, and resource-oriented cities will have a higher dependence on resources because of their unique development conditions. Human society has experienced the stage of economic and social development from actively adapting to the natural environment to plundering resources, and the short-term rapid enhancement of economic output has destroyed the ecological environment and large-scale environmental pollution at the same time. The current concept of sustainable development, especially for resource-based cities, is to gradually move towards the road of transformation, an important part of which is to improve the utilization of resources the restoration and protection of the ecological environment, and the treatment of pollution. Therefore, this paper sets the resource environment subsystem as natural resource factor (NRF) and pollution control factor (PCF), which are composed of temperature (T), precipitation (P), domestic water consumption (DWC), industrial water withdrawal (IWW), green coverage (GC), as well as the number of days with good air quality (DGAQ), harmless disposal rate of domestic garbage (HDRDW), sewage treatment rate (STR), industrial waste gas emission rate (STR), and the number of days with good air quality (DGAQ), harmless disposal rate of domestic waste (HDRDW), sewage treatment rate (STR). STR), industrial waste gas emission (IWGE), and general industrial solid waste disposal rate (ISWDR) are composed.

The correlations and mathematical methods of SD model construction resulted in mathematical equations between variables (Table 3). Through the error calculation method, it can be obtained that the average relative error of the total land use area is 0.315%, and the model accuracy is overall high^42^. Separately, the average relative errors of cropland, forest land, watershed, and construction land are less than 3%, and grassland is slightly higher than the errors of other land use, but still less than 10%, which meets the requirements of the fitting accuracy^43^, and due to the area of unutilized land is too small, the model test error is referenced to the error of the total land use area, and in summary, the model can be used to simulate the future land use changes.

**Table 3:** Table of mathematical equations of SD model of resource-based city transformation based on land use.

Systems	Equation	Number
Economic subsystems	FIO = 0.0167 * GDP + 5.8221	(1)
	PIO = 0.0112 * GDP + 6.9769	(2)
	FIO = 0.0079 * GDP − 0.2239	(3)
	AIO = 0.0264 * GDP + 11.691	(4)
	SIO = 0.002 * GDP + 1.221	(5)
	FO = 658.881 * FIO − 27.497 * TP + 110.824 * CA + 49,468.174	(6)
	MO = 2269.330 * PIO-23.783 * TP + 20,588.244 * GL + 18,306.401	(7)
	FPO = 1403.295 * AIO + 238.483 * TP − 786.932 * CL + 18,800,031.407	(8)
	IPO = 2543.123 * IO + 25.123 * TP + 56.345 * IL + 109.246	(9)
social subsystem	SQ = (MF^2^ + IF^2^ + SSF^2^ + EF^2^)^1/4^	(10)
	MF = (NMB^2^ + NHFP^2^)^1/2^	(11)
	IF = (RAPC^2^ + CGPR^2^ + CWPR^2^ + TPRP^2^ + PAP^2^)^1/5^	(12)
	SSF = (NBMIP^2^ + NUIP^2^ + MBPIP^2^)^1/3^	(13)
	EF = (GHETR^2^ + GSETR^2^ + SSTR^2^ + ESTR^2^)^1/4^	(14)
Population subsystem	TP = UP + RP	(15)
	PD = TP/LA	(16)
Science and technology subsystem	SRI = (REDS^2^ + NMSP^2^ + DRI^2^ + NPAG^2^ + LCRI^2^)^1/5^	(17)
Resource Environment Subsystem	RE = (NRF^2^ + PCF^2^)^1/2^	(18)
	NRF = (GC^2^ + IWW^2^ + DWC^2^ + T^2^ + P^2^)^1/5^	(19)
	PCF = (DGAQ^2^ + HDRDW^2^ + STR^2^ + IWGE^2^ + ISWDR^2+IWGE2+DGAQ2^)^1/7^	(20)
Land use system	TL = 2964	(21)
	CL = − 0.006 * P − 0.429 * GGR + 0.973 * AIO − 8.831 * TP − 8.986e−05 * FPO − 0.531 * UR − 0.71 * IL + 3497.18	(22)
	FL = − 0.079 * P + 0.529 * GGR − 1.958 * FIO + 8.599 * TP + 0.459 * UR − 49.74 * SF − 104.408 * NRF + 13.847 * SPF + 73.544 * GL + 1.802 * IL − 1.148 * DGAQ + 0.005 * IWGE − 23.368 * T + 82.88	(23)
	WA = − 0.181 * FL + 41.954 * GL + − 1.924 * UR − 0.007 * P + 5.095 * FIO + 9.786e − 06 * FO − 6.273 * TP + 23.28 * SF − 1.572 * SPF + 0.812 * STR + 44.99 * NRF + 0.018 * UR + 1400.27	(24)
	GL = (0.013 * IL + 0.001 * P − 0.09 * PIO + 2.331e − 05 * MO + 0.07 * TP − 0.003 * GGR + 0.012 * UR − 0.299 * SF − 0.442 * NRF − 0.157 * SPF − 0.014 * GC − 0.006 * STR − 9.58) * 0.71–0.0036	(25)
	IL = − 0.245 * GGR − 0.186 * UR + 0.11 * NAGDP + 5.568 * SF + 5.996 * PCF + 0.99 * SPF + 70.823	(26)
	BL = TL–CL–FL–GL–WA–IL	(27)

#### Analysis of the results of the land-use structure simulation

The simulation results under multiple scenarios for each land type in Tongling City were obtained based on the SD modeling method (Table 4). The land use change trend of each land use type is in line with the expectation of the development state, presenting a smooth and differentiated change characteristic. Based on the rolling forecasting basis, the indicators under different development scenarios have a time-ordered impact on land use change and each land type changes continuously with the increase of the forecasting year. The diversity and difference in land use structure are influenced by human activities, and the expansion of urban and farmland scale will lead to the reduction of the number of ecological lands and the increase of the degree of fragmentation, which brings about the land use pattern of functional imbalance^44^.

**Table 4:** Scenario simulation results table.

Contextualization	Natural trends														
Year	2021	2022	2023	2024	2025	2026	2027	2028	2029	2030	2031	2032	2033	2034	2035
a (km^2^)	1784.77	1776.53	1767.75	1755.62	1749.37	1741.52	1734.17	1726.56	1714.9	1706.38	1698.86	1690.33	1684.79	1676.42	1670.69
b (km^2^)	575.85	574.26	572.42	570.61	568.73	567.57	566.81	563.53	560.69	557.76	556.64	555.23	554.92	553.63	552.75
c (km^2^)	383.41	383.11	382.71	381.71	380.92	379.13	377.34	375.51	373.72	372.53	370.14	368.38	366.57	365.92	365.71
d (km^2^)	0.2039	0.1941	0.1842	0.1744	0.1646	0.1547	0.1449	0.1351	0.1252	0.1154	0.1054	0.0954	0.0827	0.0701	0.0673
e (km^2^)	219.61	229.06	240.74	255.05	264.67	275.01	284.85	297.44	314.03	326.22	337.31	349.01	356.69	367.63	373.89
f (km^2^)	0.000902	0.000882	0.000902	0.000852	0.000794	0.000705	0.000665	0.000622	0.000616	0.000601	0.000591	0.000581	0.000559	0.000538	0.000517

Contextualization	Economic development														
Year	2021	2022	2023	2024	2025	2026	2027	2028	2029	2030	2031	2032	2033	2034	2035
a (km^2^)	1770.36	1763.78	1755.14	1746.59	1738.04	1719.56	1707.02	1698.58	1680.08	1675.56	1670.43	1655.43	1650.82	1638.91	1620.46
b (km^2^)	572.56	570.85	567.42	562.63	560.67	557.91	556.64	555.62	554.56	552.75	551.91	549.89	548.56	546.76	545.31
c (km^2^)	382.32	380.14	378.23	376.64	374.85	371.64	368.64	367.67	365.94	363.85	361.76	359.72	356.64	354.86	352.94
d (km^2^)	0.1939	0.1841	0.1802	0.1684	0.1576	0.1447	0.1349	0.1201	0.1152	0.0954	0.0854	0.0754	0.0627	0.0581	0.0503
e (km^2^)	237.74	248.66	262.71	277.88	289.52	313.84	331.16	341.48	362.79	371.51	379.31	398.42	407.71	422.77	444.46
f (km^2^)	0.000872	0.000862	0.000852	0.000802	0.000744	0.000675	0.000635	0.000602	0.000582	0.000556	0.000539	0.000521	0.000509	0.000489	0.000465

Contextualization	Social progress														
Year	2021	2022	2023	2024	2025	2026	2027	2028	2029	2030	2031	2032	2033	2034	2035
a (km^2^)	1776.35	1767.82	1761.93	1750.64	1742.76	1728.55	1718.26	1708.78	1694.56	1686.54	1674.29	1665.43	1658.55	1649.57	1634.63
b (km^2^)	574.31	572.03	568.05	566.65	563.42	562.76	560.02	558.36	556.43	554.79	552.85	550.53	549.24	548.61	546.27
c (km^2^)	382.605	381.105	379.705	379.105	376.905	375.105	373.305	370.505	368.705	364.505	363.105	362.305	360.505	358.925	356.715
d (km^2^)	0.1989	0.1891	0.1822	0.1714	0.1611	0.1497	0.1398	0.1276	0.1202	0.1054	0.0954	0.0854	0.0727	0.0641	0.0588
e (km^2^)	230.24	242.36	253.51	267.28	280.32	296.64	311.96	325.28	343.39	357.32	373.02	385.11	395.41	406.47	426.16
f (km^2^)	0.000887	0.000872	0.000877	0.000827	0.000769	0.000692	0.000653	0.000612	0.000599	0.000578	0.000565	0.000551	0.000534	0.000513	0.000491

Contextualization	Resource and environmental protection														
Year	2021	2022	2023	2024	2025	2026	2027	2028	2029	2030	2031	2032	2033	2034	2035
a (km^2^)	1788.74	1780.53	1769.62	1759.45	1752.36	1743.52	1737.17	1729.68	1717.94	1709.32	1700.84	1695.39	1688.81	1684.45	1675.91
b (km^2^)	576.52	575.64	573.81	571.73	569.85	568.67	567.84	564.64	562.91	558.36	557.76	556.57	555.59	554.75	553.94
c (km^2^)	383.54	383.91	383.76	382.34	381.53	380.43	378.61	376.76	374.91	373.52	371.61	369.72	366.65	366.89	367.76
d (km^2^)	0.2139	0.2041	0.1942	0.1854	0.1716	0.1627	0.1632	0.1571	0.1382	0.1304	0.1154	0.1054	0.1027	0.0931	0.0973
e (km^2^)	214.64	223.49	235.63	249.69	259.11	270.39	279.56	291.98	307.59	322.21	332.91	342.01	351.51	357.07	366.06
f (km^2^)	0.000922	0.000902	0.000922	0.000872	0.000824	0.000755	0.000695	0.000643	0.000635	0.000628	0.00062	0.000608	0.000584	0.000574	0.000562

Contextualization	Coordinated development														
Year	2021	2022	2023	2024	2025	2026	2027	2028	2029	2030	2031	2032	2033	2034	2035
a (km^2^)	1780.05	1772.16	1763.61	1753.07	1745.63	1733.28	1724.15	1715.91	1701.87	1694.45	1686.11	1676.65	1670.74	1662.33	1650.42
b (km^2^)	574.81	573.19	570.42	567.91	565.66	564.22	562.82	560.53	558.64	555.91	554.79	553.05	552.07	550.93	549.56
c (km^2^)	382.96	382.06	381.11	379.94	378.55	376.57	374.47	372.61	370.82	368.62	366.65	365.03	362.59	361.64	360.78
d (km^2^)	0.2026	0.1928	0.1852	0.1749	0.1637	0.1529	0.1457	0.1349	0.1247	0.1116	0.1004	0.0904	0.0802	0.0713	0.0684
e (km^2^)	225.71	236.07	248.52	262.44	273.06	288.96	301.46	313.83	331.69	343.96	355.66	368.76	378.12	388.54	402.94
f (km^2^)	0.000895	0.000872	0.000888	0.000838	0.000782	0.000706	0.000662	0.000619	0.000608	0.000591	0.000572	0.000565	0.000546	0.000585	0.000508

a, b, c, d, e, and f represent cultivated land, forest land, grassland, water area, construction land, and unused land.

From the perspective of land category difference, cropland is the land category with the largest decrease in the change difference, and according to the prediction of the five scenarios, the rate of change to 2035 is − 6.44%, − 9.25%, − 8.46%, − 6.15%, and − 7.58%, respectively, which shows a large development difference. Among them, the ED scenario has the largest difference in the area from the PRE scenario, which is 55.45 km^2^, and the difference in the rate of change is 3.1%. The water and forest change differences are the next most decreasing. The extreme difference in the area changes of water to 2035 is 14.82 km^2^, while the extreme difference in the rate of change of forest is 1.5%. Grassland and barren have fewer values of area change due to their smaller overall area, but the rate of change is relatively larger, and the extreme difference in the rate of change for the two types of land is 27% and 15.75%. Impervious has the opposite direction of change to the above five types of land and the largest change in the area, with an area of 216.29 km^2^ in 2020, and the extreme difference in the rate of change of contextual differences in 2035 is 36.25%.

From the contextual difference perspective, the NT scenario evolves in each category based on land use change in historical years. The rate of change in cropland remains between − 3% and − 5% in most years, with faster deceleration in 2023–2024 and 2028–2029, when the rate of change exceeds − 11%. Forest decreases at a rate of more than -0.5% in 2027–2030, and water decreases more in 2025–2033 than in other years. Grassland and barren have a small base and do not change significantly in quantity. Impervious maintains its growth pattern, growing faster in the 2022–2024 and 2027–2032 phases, at a rate of more than 5%. Impervious grows the fastest in the ED scenario, while cropland, forest, and water all show a significant downward trend. In particular, the rate of decline of cropland increases every five years, approaching − 3% between 2031 and 2035, while the rate of decline of forest, water, and grassland is relatively flat and stable. Impervious will increase by more than 17% every five years, with the most prominent year-to-year increases in 2025–2026 and 2028–2029. The level of regional economic development is inextricably linked with the degree of urbanization and population agglomeration, and in this context, the impacts of land-use structure have received the attention of scholars. Wanxu Chen et. al. found that in regions with higher economic development levels, the rate of growth of land-use intensity and land-use structure change is more obvious^45^. Xiaoqing Song et al. concluded that the higher the population density, the more land allocation will be biased towards secondary and tertiary industries, thus bringing land-use structure use offset^46^. This is consistent with this study’s finding that the increase in construction land use is higher in the economic development context than in other contexts.

In the SP scenario, the deceleration of cropland, forest, and water is relatively slower than in the ED scenario, but the overall deceleration of cropland and grassland in 2021–2025 and of forest and water in 2026–2030 is faster than that of ED scenario in the SP scenario. The growth rate of Impervious is also relatively controlled, especially slowing down to 14.25% in 2031–2035. The social system is a complex giant system, which contains many elements and spaces that measure the production and life of human beings. Zhenhua Chen et al. explored the impact of high-speed rail development on land use structure from the perspective of high-speed rail development and came to the conclusion that the development of transportation infrastructure has a differential impact on the land use structure in different urban scales^47^. The area of land categories such as cropland, forest, grassland, and water under the RED scenario is higher than the historical development trend over the same period, while the area of impervious under this scenario is lower than the historical development trend over the same period. In terms of the overall trend of change, the inter-annual rate of change is low, with cropland less than − 2%, forest and water remaining at around − 1%, and the growth rate of impervious being controlled within 20% in every five years, especially the growth rate of 2031–2035 is reduced by − 0.88%, − 4.28%, and − 7.22% compared to that of NT, SP, ED respectively. In the context of coordinated development, the land-use structure has been developed in a balanced manner. The growth of construction land is limited, and under the premise of guaranteeing food security carrying the land type of arable land, the ecological functional land types such as forest land, grassland, and water area are reasonably allocated. Optimization of land use structure can not only balance the relationship between economic benefits and ecosystem value but also make the transformation of resource cities in the new period in line with the law of industrial structure optimization, to achieve the goal of coordinated development^48,49^.

### Land use layout simulation analysis

#### Model setup and accuracy check

**Figure 5:**
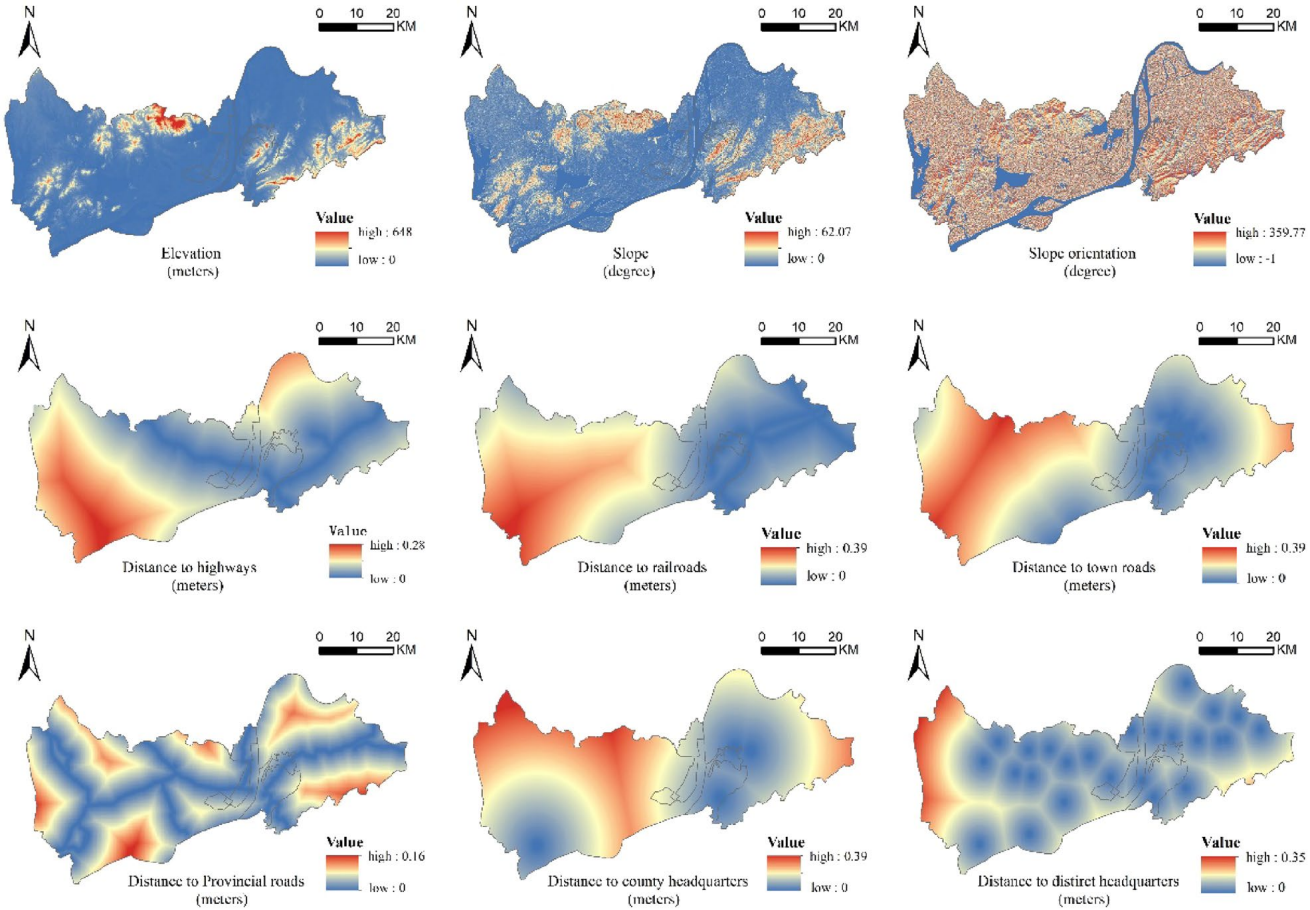
Driving factors of territorial spatial layout simulation in Tongling. *Note*: Revision number is GS (2020)4619, no modifications to the base map, URL: http://bzdt.ch.mnr.gov.cn/index.html. This diagram was created by the software GIS 10.6, URL: https://desktop.arcgis.com/zh-cn/index.html.

Referring to the experience of existing studies and considering the land use characteristics of the study area, considering the current situation of Tongling, the parameters of the neighborhood factors are defined as follows (Table 5). Natural factors, transportation conditions, and geographical proximity are the basis of resource endowment, spatial layout, and expansion of a region. Referring to related studies, the spatial drivers of this paper are set as follows: elevation, slope, slope direction, distance from a highway, distance from railroad, distance from highway, distance from provincial highway, distance from municipal government, distance from county (district) government (Fig. 5). According to different functional improvement scenarios, set the conversion levels for different types of land. Based on the SD modeling scenario rules, five types of conversion cost matrices corresponding to them are set (Table 6).

**Table 5:** Neighborhood factor parameters.

Land use type	Farmland	Forest land	Grassland	Water land	Unused land	Construction land
*w*_*k*_ value	0.5	0.1	0.08	0.3	0.02	1

**Table 6:** Conversion cost matrix.

Contextualization	Conversion cost						
Natural trends	Land use type	a	b	c	d	e	f
	a	1	1	0	0	0	1
	b	0	1	0	0	0	1
	c	0	1	1	0	0	1
	d	0	1	0	1	0	1
	e	1	1	1	1	1	1
	f	0	1	0	0	0	1
Economic development	Land use type	a	b	c	d	e	f
	a	1	1	1	1	0	1
	b	1	1	1	1	0	1
	c	1	1	1	0	0	1
	d	1	1	1	1	0	1
	e	1	1	1	1	1	1
	f	0	0	0	0	0	1
Social progress	Land use type	a	b	c	d	e	f
	a	1	1	1	1	0	1
	b	0	1	1	1	0	0
	c	0	1	1	1	0	0
	d	0	1	1	1	0	1
	e	1	1	1	1	1	1
	f	0	0	0	0	0	1
Resource and environmental protection	Land use type	a	b	c	d	e	f
	a	1	1	1	1	0	0
	b	0	1	1	1	0	0
	c	0	1	1	1	0	0
	d	0	1	1	1	0	0
	e	1	1	1	1	1	1
	f	1	1	1	1	0	1
Coordinated development	Land use type	a	b	c	d	e	f
	a	1	1	1	1	0	1
	b	0	1	1	1	0	1
	c	0	1	1	1	0	1
	d	0	1	1	1	0	1
	e	1	1	1	1	1	1
	f	0	0	0	0	0	1

a, b, c, d, e and f represent cultivated land, forest land, grassland, water area, construction land, and unused land, respectively; 1 means it can be converted, and 0 means it cannot be converted.

To ensure that the prediction results are scientific, reasonable, and in line with reality, it is necessary to verify the simulation accuracy. The Kappa coefficient, as an important index coefficient for analyzing the consistency of remote sensing images, can be used to carry out spatial simulation models based on the verification of the accuracy of the model. The closer the value of the Kappa coefficient is to 1, the higher the simulation accuracy is, and when the Kappa coefficient is greater than 0.8, it indicates that the model simulation accuracy can be applied^50^. In this paper, through the accuracy calculation of the test data and the actual data, we get that the Kappa coefficient of this validation is 0.987, the simulation accuracy is good, and the model simulation can be carried out.

#### Analysis of land use layout simulation results

**Figure 6:**
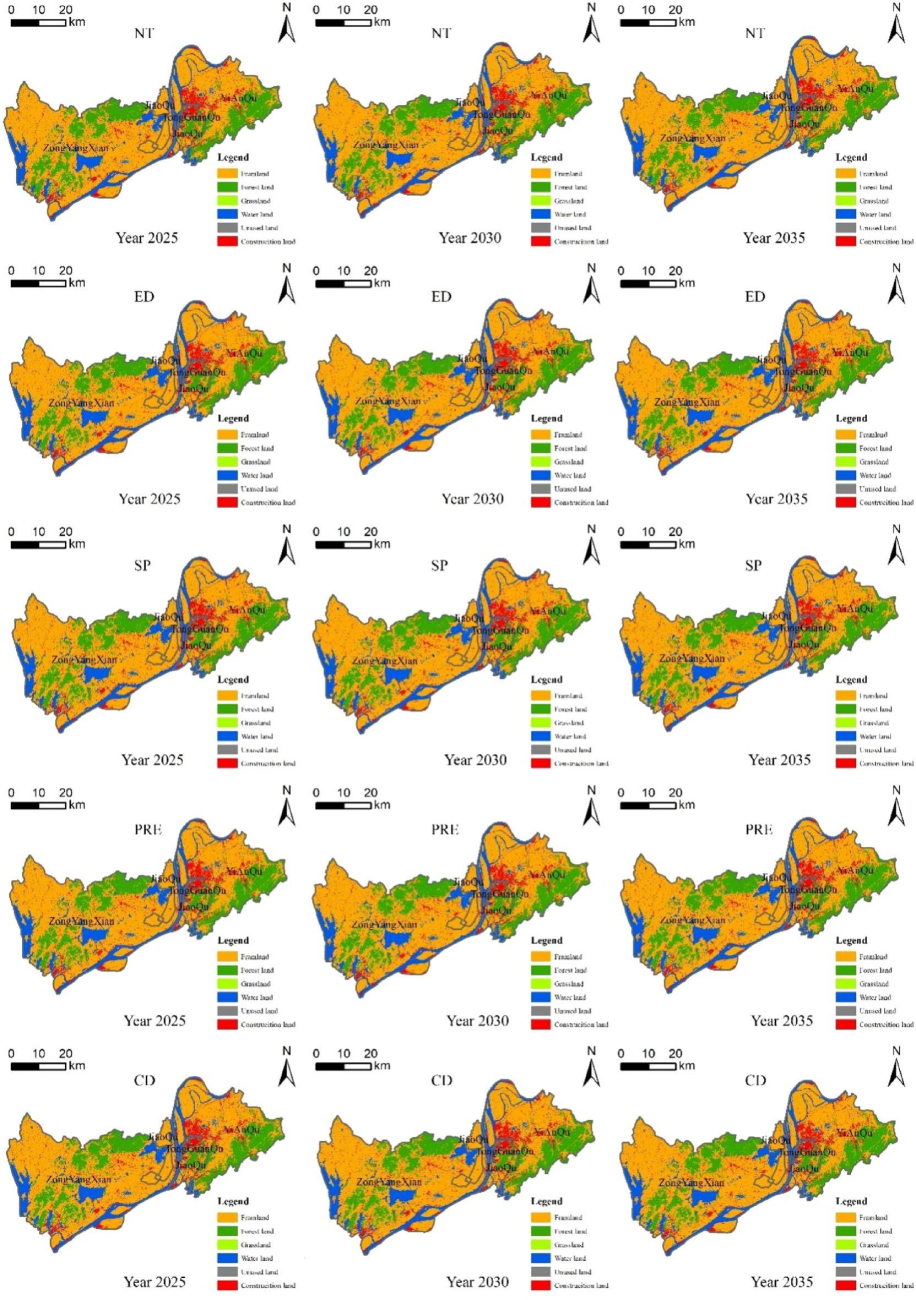
Situational simulation of Tongling’s land. Note: Revision number is GS (2020)4619, no modifications to the base map, URL: http://bzdt.ch.mnr.gov.cn/index.html. This diagram was created by the software GIS 10.6, URL: https://desktop.arcgis.com/zh-cn/index.html.

Based on the SD model results and applying the FLUS modeling approach, the layout results for 2025, 2030, and 2035 were derived for the five scenarios (Fig. 6). From the contextual difference perspective, the NT scenario evolved for each class based on land use changes in historical years. The rate of change in cropland stays between -3% and -5% in most years, with faster deceleration in 2023–2024 and 2028–2029, when the rate of change exceeds -11%. Forest decreases at a rate of more than − 0.5% in 2027–2030, and water decreases more in 2025–2033 than in other years. Grassland and barren have a small base and do not change significantly in quantity. Impervious maintains its growth trend, growing faster in the 2022–2024 and 2027–2032 phases, at a rate of more than 5%. CD scenario is a model condition set up to balance the relationship between ED, SP, and PRE scenarios. The rate of change of cropland is around − 2% every five years, and the deceleration is smaller than the ED and SP scenarios in 2021–2025, and smaller than the PRE scenario in 2026–2035. Forest decelerates less than the ED and SP scenario and more than the PRE scenario in 2026–2030. The rate of change in water is greater than in the ED and SP scenarios, but less than in the RPE scenario. The overall trend of grassland is similar to that of cropland. The rate of change of impervious in this scenario is still within 20% every five years, and the overall growth rate is smaller than that of the SP scenario, and the growth rate in 2026–2030 is larger than that of the ED scenario but smaller than that of PRE scenario. The method of setting up scenarios with different focuses to simulate future land use patterns based on different development goals of each region is widely used. For example, Qiang Huang et al. focus on the hollowing out of rural settlements and try to explore a layout that reduces the loss of agricultural land, protects the ecological environment, and improves the conditions of agricultural production and the living standards of rural villagers^51^. Yingchun Liu et al. focus on how to promote Watershed protection and land use layout optimization in Southwest Lake Basin, China^52^.

From the perspective of spatial layout, Tongling’s cropland is concentrated and contiguous, mainly located in the northwest and southeast of Zongyang County and the north-central part of Yian District, where the terrain is relatively flat and the irrigation conditions are excellent, which is suitable for the development of agricultural farming, and therefore will show a trend of in-situ retention and quality improvement. Forest is currently located mainly in the north and southwest of Zongyang County and the southeast of Yian District, and future reductions will be concentrated in small areas in the north of Zongyang County and the southeast of Yian District. In addition to the Yangtze River water system, which flows in a southwestern-northeastern direction, which will be preserved and protected under the principle of national strategic protection, a small part of the Tong guan-Yian agglomeration will be expanded to ensure quality and livable living for the residents, and a part of Zongyang County will be given over to the expansion of land for construction under the goal of the new county development. Impervious will remain concentrated in the areas of the former Tong guan and Yian Districts where the population and economic activities are most active and will expand and extend to the northeast. To enhance the economic development focus of Zongyang County and promote industrial restructuring, the southwest corner of the former county will undergo a certain degree of outward expansion. The trend of changes in the regional spatial layout is not only guided or limited by the natural endowment conditions of the region, such as topography and geomorphology but also affected by many other factors. Among them, it is very important to clarify the suitability of spatial layout. To alleviate the conflict between economic development and the ecological environment, exploring the coordinated symbiosis of production, ecology, and living space is an important path to promote sustainable development^53^. Strengthening the construction of green infrastructure can also present a certain buffer role for cities to withstand natural disasters^54^. The expansion trend of urban construction land in highly urbanized areas has intensified, and the phenomenon of idleness has been highlighted. To enhance land use intensification and ensure the rational allocation of ecological space, Hong et al.^55^ proposed the concept of construction land clearance, which provides new ideas for the disposal of construction land stock.

## Discussions

### Exploration of transformation trends in resource-based cities

Based on the above exploration of the transformation and development of Tongling the future development direction of Tongling is further clarified. At the same time, the experience of transformation and development can be extracted to provide path thinking and ideas for the transformation and development of the same type of cities. Tongling’s over-reliance on industry in the previous stage of economic development has brought about a certain degree of imbalance in the industrial structure, but it can rely on development to improve the level of copper mining, smelting, and deep processing. For the time being, copper products are still an important accessory in many fields of national economic construction, such as aerospace, infrastructure engineering, modern communications, etc.^56^. But should abandon or gradually replace the primary copper product processing, to have a high quality, high level of processing industry. In recent years, the layout of fixed asset investment in Tongling has gradually shifted from the traditional mining industry to the information transmission, software and information technology services, financial industry, scientific research, and technology services, etc., which lays a certain foundation for the adjustment of the industrial structure and puts into practice the scholars’ theoretical viewpoints on the selection, optimization, and transformation of the regional pillar industries^57^.

It is worth mentioning that Tongling combines natural scenery with unique humanistic features in an attempt to create a cultural tourism brand. For example, “Jin-niu-dong Ancient Mining Ruins” began in the Spring and Autumn Period, with the important historical value of studying China’s bronze culture and the history of mining and metallurgy. Since 1992, the municipal government has strengthened the protection and restoration of the work, and the construction of open scenic spots, in an attempt to promote the dissemination of the bronze culture, for the transformation of the city of Tongling development opens up a new direction. Looking at the transformation path of the global resource-based cities, there can be the development of a cultural tourism industry matrix. Reference can be made to Tongling, Daqing City, Heilongjiang Province, the construction of the mining historical sites park, oil extraction culture park. Reference can be made to Huainan City, Anhui Province, coal mining subsidence area for ecological restoration, and the construction of ecological parks with the function of rest and recuperation. Reference can also be made to the city of Essen in the western German state of North Rhine-Westphalia, which has taken advantage of its vast forests and lakes to build a local leisure and service center.

Many resource cities are trapped in upgrading the product technology and processing level^34^, but to adapt to the precision instruments, emerging equipment, and modern communications, Tongling has upgraded its scientific research level, cooperated closely with colleges and universities, constructed high-level scientific research institutes, increased its independent R&D investment, and set up a large-scale public R&D platform, mastering more core technologies and original products, which reflects the current transformation of the resource cities relying on the scientific and technological progress. This reflects the rationality and necessity of the current transformation of resource cities relying on scientific and technological progress. At the same time, modern logistics and modern finance have gradually become the key links in the development of various industries, especially the development of resource-based real industries. Tongling has strengthened port construction, relied on logistics networks to promote product exchanges, and built a financial platform to facilitate trade cooperation, which has become the follow-up power for transformation and development. The government departments of resource-based cities should not only favor the emerging industries of science and technology in fixed asset investment but also support the improvement of science and technology to ensure the sustainable vitality of the city’s transformation.

Previously, the ecological damage and environmental pollution caused by the development of resource-based cities have been criticized^58^, and the policy requirement of carrying out comprehensive ecological environment management was put forward in the *Fourteenth Five-Year Plan for Promoting High-Quality Development of Resource-Based Areas* issued by China in 2021, which demonstrates the importance of the construction of ecological civilization for the development of resource-based cities^59^. In recent years, Tongling has formed an eco-industrial chain around the comprehensive utilization of mineral resources, and actively constructed the Riverside Circular Economy Industrial Pilot Park and the Heng-gang Circular Economy Industrial Demonstration Zone, to enhance industrial concentration, improve the efficiency of energy utilization, strengthen the level of pollution control, and promote the green transformation of enterprises, so that the ecological environment has been effectively improved, and the level of livability of the residents has been vigorously enhanced. Under the development goal of ecological civilization, the transformation of resource cities should pay more attention to the protection of resources and environment, implement the investigation of natural resources, strengthen the level of ecological restoration, and help to promote the realization of the dual-carbon goal.

Population and labor force are the important cornerstones of urban development, and if resource cities want to have sustainable development, not only stabilize, and promote employment, but also attention to the impact of the level of public services and basic livelihood security on the lives of the population. Resource-based cities are at risk of large-scale unemployment due to resource depletion and industrial fluctuations, so they should not only do a good job in compensating laid-off workers for unemployment but also provide vocational skills training to improve the level and quality of re-employment. At the same time, in the process of resource-based cities gradually shifting from serving resource-based industrial development to coordinated development, the level of urban infrastructure construction and public service guarantee needs to be vigorously upgraded concerning safeguarding the living standard of the residents.

### Exploring the optimization of land spatial patterns in the transformation process of resource-based cities

Territory spatial pattern is the overall layout of the integrated space of natural resources, human life, economic environment, social development, etc., while the optimization of territory spatial pattern is based on the worldwide thinking of sustainable development, and it is proposed to promote the holistic construction of spatial planning and the coordinated development of each element based on territory space. The structural optimization and spatial layout optimization of territory space optimization complement one another and promote the rational and efficient spatial layout under the premise of rational allocation of the quantity and structure of each land use.

The simulation study on structural optimization in this paper shows that there are obvious differences in the magnitude of change of each site under different development goals. When economic development is the main goal, the output per unit area of land is an important criterion, and the industry carried by the construction land can realize this goal, so the number of land structure changes brought about by the expansion of the construction land area can be satisfied by the reduction of the area of other land. However, as the country’s requirements for the quality of economic development have been raised, Tongling governments at all levels are also striving to promote the simultaneous development of new industrialization, informatization, urbanization, and agricultural modernization, and to promote the linked development of the north and the south of Yangtze River, to effectively improve the quality and level of urbanization development. To guarantee national food security, the red line of 1.8 billion mu of arable land will not be breached, highlighting the ecological protection of the Yangtze River, delineating the red line of ecological protection, and strengthening ecological restoration and other objectives have been put forward, and the rate of expansion of construction land has been effectively controlled. In this paper, the setting of the PRE scenario is to focus on the protection of cropland, forest water, grassland, and other land with ecological functions that will not be arbitrarily converted to use, so that the increase in the area of impervious has been reduced compared with other development scenarios. Resource-based cities have some commonalities compared with other cities, so they are in line with the consensus law of impervious expansion brought about by the process of urban development^60^. However, the history of resource-based development laid the foundation of the city at the same time brought the problems of industrial development imbalance, ecological damage, and environmental pollution, in the process of transformation and adjustment, downsizing and expansion of the scale of the primary industry and industry types, repairing the damaged mountains, geology, purifying pollution sources, controlling pollutant emissions, improving the city’s comprehensive service functions, and safeguarding the production and life of urban residents.

After the quantity and structure of each site are reasonably allocated, the spatial layout should be further optimized to reflect the harmonious and mutually reinforcing spatial relationship of each site. The Tongling government’s development concept includes the overall goal of promoting the linkage development of the north and the south of the Yangtze River, as well as the hierarchical development goal of constructing a spatial system of “one belt, three corridors, three layers, and five levels”. From the results of the above simulation of Tongling’s land use layout, it can be seen that the spatial change trend of land types is mainly based on the original site for certain expansion or downsizing. To further promote the transformation process and enhance the optimization of the spatial layout of Tongling City, all regions at all levels should respond to the layout and transformation based on the main functions and development characteristics. Tong guan District, as the center of Tongling City, is responsible for important social and economic functions, with a relatively high concentration of population and industry, and is dominated by construction land. Therefore, it is necessary to coordinate the layout of construction land in the district and focus on urban villages and old neighborhoods to improve living conditions. It is necessary to make up for the facilities around the commercial and service industry centers, strengthen the pollution emission monitoring of copper industry processing enterprises and the supervision of park utilization, carry out soil and water conservation for the forest land in the district and water source protection for urban wetlands, and enhance the efficiency of space utilization. Yian District, adjacent to Tong guan District, should rely on the development foundation of the city’s sub-center, cluster and enhance the residential and construction areas in the district, carry out comprehensive improvement of woodlands on low-slope hills, protect the water quality of the Yangtze River with the leading role of the Riverside Eco-Park, develop high-quality agriculture relying on the advantages of the topography and landscape of the river rambling beach in the middle and lower reaches of the Yangtze River, and develop the regional transportation to create a new shipping center relying on the Hu-Yu Expressway crossing the border and the Yangtze River’s water transportation advantages. Center. Suburban industrial parks are clustered and are important industrial development zones. Accelerate the promotion of the Economic Development Zone to the north of the river transfer area, committed to creating a “port, industry, and city integration” of the new city north of the river Port. At the same time to pay attention to the Yangtze River waters environmental testing, environmental impact assessment, soil and water conservation, water resources demonstration, and other six regional assessments, the development and protection of a combination. Zongyang County is an important area for cross-river development in Tongling. High-quality and high-level agriculture will be developed based on agricultural development in the county and district, and a production, processing, and supply base will be built. The forest land concentration areas in the north and southwest of the county will be strengthened with soil and water remediation, connecting wetlands and lakes of the Yangtze River system, and realizing ditches and canals to prevent and mitigate floods. Relying on the advantages of forest and water systems in the county to create a tourism brand. Resource-oriented cities in the spatial optimization of the layout adjustment process, not only to protect the advantageous location of pillar-type industries but also with the new stage of traffic development, port construction, and urban expansion of the current situation. At the same time, pay attention to the hierarchical layout of the spatial structure, the original spatial agglomeration of urban functions for the secondary decomposition, around the advantages of different regions for the layout of the main functions, to alleviate the pressure of the original central agglomeration area.

### Prospects

First, this paper chooses economy, society, population, science and technology, resources, and environment as the important aspects for judging the transformation and development of Tongling and combines the selection of indicators to set up a differentiated situation and construct the relationship equation of land use structure, to simulate the quantitative structure and spatial layout of land use, which is systematic to a certain extent. However, the selection process of indicators is highly subjective, and there are still a lot of influencing factors in the transformation process, so it is necessary to define the indicators further scientifically for scientific prediction. Secondly, this paper selects six indicators, namely, GDP growth rate, urbanization rate, social factor, natural factor, pollution control factor, and scientific and technological progress factor, and sets up NT, ED, SP, PRE, and CD five scenarios for the simulation of land use of resource cities, which reflects the differences of the scenarios to a certain extent. However, there are still a variety of combination possibilities for the setting of scenarios, so it is possible to further set up comprehensive scenarios based on the current situation, to be closer to the actual development. In future research, there are still directions to be expanded: (1) supplementing the index elements to build a more scientific and reasonable analytical framework; (2) expanding the research perspective to include the impacts of government policies in the logical framework; (3) broadening the research object by selecting similar prefectures from a certain regional scope, and studying their development history, current situation, and future trends to compare and derive the development differences.

## Conclusions

This paper takes Tongling City as a piece of evidence to sort out the ideas of transformation and development, based on the development objectives proposed by the relevant departments, using land use, spatial variables, statistics, etc. to obtain the relevant data of Tongling City from 2005 to 2020, and using the methods of literature analysis, spatial analysis, and model analysis to simulate the structure and layout of the land use in Tongling City, as well as to analyze the trend of the transformation and the optimization of the layout of homeland space scheme. The specific conclusions are as follows:

1. As a systemic concept, the transformational development of resource cities constitutes a hierarchical framework of “system-indicator-element”. It is influenced by economic, social, demographic, scientific and technological, and resource and environmental subsystems, with GDP growth rate, quality of social development, population, level of social progress, and level of resource and environmental conditions being the characterizing indicators, and the specific elements of which are not only interconnected and inextricably linked but also interact with each other and with different land types to influence the land-use system.
2. Simulation results of land use structure prediction based on SD model construction. Overall, the development of contextual trends of land categories is relatively similar, the decrease of cropland, forest, water, grassland, and barren decreases from ED, SP, and CD scenario, and the area has been slightly increased under the scenario of PRE. On the contrary, the increase of construction land decreases from ED, SP, and CD scenarios, and the expansion of land area is controlled to a certain extent in the PRE scenario. From the viewpoint of land classification, there is a significant difference in the land use changes under different scenarios.
3. Layout simulation results for Tongling in 2025, 2030, and 2035 under five scenarios are derived based on the FLUS model. Cultivated land accounts for a large proportion of the total land area and is mainly distributed in Zongyang County and the eastern part of Yian District, forest is mainly located in the north and southwest of Zongyang County and the southeast of Yian District, and there are lakes in Zongyang County in addition to the Yangtze River system in the water. Impervious has the highest density in Tong guan District, followed by the area bordering Tong guan District in the west of Yian District, with a small concentration in the south-west corner of Zongyang County.

## Data Availability

The data supporting the findings of the study are available from the first author upon request.
